# The Neural Mechanism by Which the Dorsal Vagal Complex Mediates the Regulation of the Gastric Motility by Weishu (RN12) and Zhongwan (BL21) Stimulation

**DOI:** 10.1155/2013/291764

**Published:** 2013-05-30

**Authors:** Hao Wang, Guo-ming Shen, Wei-jian Liu, Shun Huang, Meng-ting Zhang

**Affiliations:** ^1^Institute of Integrated Chinese and Western Medicine, Anhui University of Traditional Chinese Medicine, Hefei, Anhui 230038, China; ^2^Department of Thoracic Surgery, The First Affiliated Hospital of Anhui University of Traditional Chinese Medicine, Hefei, Anhui, China; ^3^Clinical College of Integrated Chinese and Western Medicine, Anhui University of Traditional Chinese Medicine, Hefei, Anhui, China

## Abstract

A large number of studies have been conducted to explore the mechanism of Back-Shu and Front-Mu points. While several lines of evidence addressed the acupuncture information of Shu acupoints and Mu acupoints gathering in the spinal cord, whether the convergence is extended to the high centre still remains unclear. The study selected gastric Mu points (RN12) and gastric Shu points (BL21) regulating gastric motility and its central neural mechanisms as the breakthrough point, using the technique of immunochemistry, nuclei lesion, electrophysiology, and nerve transection. Here, we report that gastric motility regulation of gastric Shu and Mu acupoints and their synergistic effect and the signals induced by electroacupuncture (EA) stimulation of acupoints RN12 and RN12 gather in the dorsal vagal complex (DVC), increasing the levels of gastrointestinal hormones in the DVC to regulate gastric motility through the vagus. In sum, our data demonstrate an important role of DVC and vagus in the regulation of gastric motility by EA at gastric Shu and Mu points.

## 1. Introduction

RN12 is the gastric Front-Mu point, while BL21 is the gastric Back-Shu point. The distribution of Back-Shu and Front-Mu points in the body has two characteristics: that is Yin and Yang in opposition and the other is, they are close to the Zang-fu viscera. The Back-Shu points are distributed in the back and waist, belonging to Yang, while the Front-Mu points are distributed in the chest and abdomen, belonging to Yin. The distribution of the two depends on the anatomic positioning of the viscera. In particular, the Front-Mu points are closer to the viscera, forming a relationship of Front-Mu points-viscera-Back-Shu points. According to the principles of traditional Chinese medicine, acupuncture at gastric Shu and Mu points can regulate gastric functions. Further, clinical practice demonstrates that EA at acupoints RN12 and BL21 can treat gastrointestinal (GI) diseases [1]. However, the mechanism(s) by which stimulation of RN12 and BL21 regulates gastric motility through central nuclei, as well as the neural mechanism employed in this process, still remain(s) unclear. Addressing this question can provide valuable clues for the development of effective therapeutics against gastrointestinal motility disorders.

 The DVC is composed of the dorsal motor nucleus of the vagus (DMV) and the nucleus of the solitary tract (NTS), which, respectively, contain neurons providing vagal efferent innervation to the major portion of the gastrointestinal tract and neurons receiving vagal afferent input from the viscera [2, 3]. Therefore, the DVC is considered to be a parasympathetic preganglionic center regulating gastrointestinal functions. Previous studies indicated that the DVC plays very important roles in the modulation of gastric motility by acupuncture [4, 5]. Neurophysiological studies have demonstrated that modulation of gastric motility is dependent on an intact vagus [6–8].

 In this study, we performed EA stimulation of the gastric Front-Mu points (RN12) and gastric Back-Shu points (BL21) to investigate the regulation of gastric motility as well as the neural mechanisms mediating this effect. Our study centered on the DVC-vagus nerve-gastric channel, using immunohistochemistry to monitor the expression levels of *c*-*fos*, MTL, and GAS in the DVC. Employing the techniques of central nuclei lesion and peripheral nerve transection, we endeavored to verify the central target and neural channel mediating the regulation of gastric motility by EA at RN12 and BL21. 

## 2. Materials and Methods

### 2.1. Materials

The following software, equipment, and reagents were employed in this study: PowerLab 8/30 data acquisition system; LabChart software; lesion-making device; double digital stereotaxic apparatus; anatomic microscope; self-made balloon; SDZ-IV type electronic acupuncture instrument of Hua Tuo brand; acupuncture needles; immunohistochemical kit; polyclonal rabbit primary antibody; and anti-rabbit secondary antibodies.

### 2.2. Animals and Groups

Male Sprague-Dawley rats weighing between 250~300 g were housed under controlled conditions (22~24°C, light on from 6:00 a.m. to 6:00 p.m.) with free access to food and water. They were randomly divided into the following groups: MOD group (induced gastric distention), RN12 group (EA at RN12), BL21 group (EA at BL21), RN12 + BL21 group (EA at RN12 plus BL21), vagotomy group (cutting of the bilateral subdiaphragmatic vagus), vagotomy + EA group (cutting vagus + EA at RN12 plus BL21), DVC lesion group (damaged DVC), and DVC lesion + EA group (damaged DVC + EA at RN12 plus BL21). Before each experiment, the animals were deprived of food for 18 h. All experimental protocols have been approved by the Committee of Animal Use for Research and Education of China and conformed to the National Institute of Health Guide for the Care and Use of Laboratory animals.

### 2.3. Electroacupuncture

EA stimulation was delivered via a pair of acupuncture needles at left BL21 and RN12 (BL21 is located 5 mm on the side of the twelfth thoracic vertebra, with needle electrodes inserted to a depth of 4 mm into the skin; RN12 is located on the median line of the upper abdomen, 20 mm above the umbilicus, with needle electrodes inserted to a depth of 2 mm into the skin). These needles were connected to an SDZ-IV type electronic acupuncture instrument. The frequency switched back and forth between 20 Hz and 100 Hz; the current intensity was set at 2–2.5 mA.

### 2.4. Measurement of Intragastric Pressure

Intragastric pressure was measured with the use of a rubber balloon that was tied around a polyethylene tube (PE 160) and inserted into the body of the stomach through a small incision in the duodenum. The balloon was secured at the pylorus with a suture to avoid movement, and the tube was connected to a pressure transducer (MLTO380), which was connected to a bridge amplifier of the PowerLab 8/30 system. The balloon was filled with water at 37°C (1.5–2.0 mL, the volume determined to be necessary to induce an intragastric pressure). Pressures were recorded and analyzed by LabChart, the data acquisition system, for online analysis. The exact location of the balloon was verified after each experiment.

### 2.5. DVC Lesion

The animal was deeply anesthetized with chloral hydrate and placed in a stereotaxic frame; the dorsal surface of the brain stem was then exposed. We placed the electrode tip in the DVC using the coordinates of Paxinos and Watson, and the electrode was advanced into the DVC (coordinates: *Ap* 11.3–14.3 mm, *L* 0.7–1.7 mm, and *H* 7.5–8.7 mm); the bilateral DVC was destroyed with a DC current (2 mA, 10 s) by a lesion-making device.

### 2.6. Bilateral Subdiaphragmatic Vagotomy

To demonstrate that DVC acts via stimulation of the vagal pathways, acute bilateral subdiaphragmatic vagotomy was performed: a midline incision was made in the abdominal wall, and the stomach was carefully manipulated to expose the esophagus. The subdiaphragmatic vagal trunks were exposed halfway between the diaphragm and the gastric cardia. Both anterior and posterior trunks of the vagal nerves were transected.

### 2.7. Brain Tissue Preparation

The brains were postfixed for 24 h at 4°C in the same fixative. Paraffin sections (5 *μ*m) were cut at the interaural levels of −4.24 to −5.08 mm (DVC) according to the atlas of Paxinos and Watson.

### 2.8. Immunohistochemical Analysis of *c*-*fos*, MTL, and GAS

Briefly, the brain sections were rinsed in PBS and incubated with 0.3% H_2_O_2_ for 30 min to remove endogenous peroxidase activity. All of the sections were incubated for 24 h at 4°C with the corresponding polyclonal rabbit antibody diluted in PBS containing 0.1% sodium azide and 0.3% Triton X-100 (PBS-T, pH 7.4). Following this step, sections were then rinsed in PBS and incubated for 1 h at room temperature with biotinylated goat anti-rabbit secondary antibody. Finally, brain sections were processed using the standard biotin-avidin-horseradish peroxidase method. *c*-*fos*, MTL, and GAS immunoreactivity was detected as a dark brown nuclear staining.

### 2.9. Statistical Analysis

All values were expressed as mean ± SE. Statistical analysis was performed with one-way ANOVA and LSD tests. A difference with a *P* value < 0.05 was considered statistically significant.

## 3. Results

### 3.1. The Effect of EA at RN12 and BL21 on IGP

**Figure 1:**
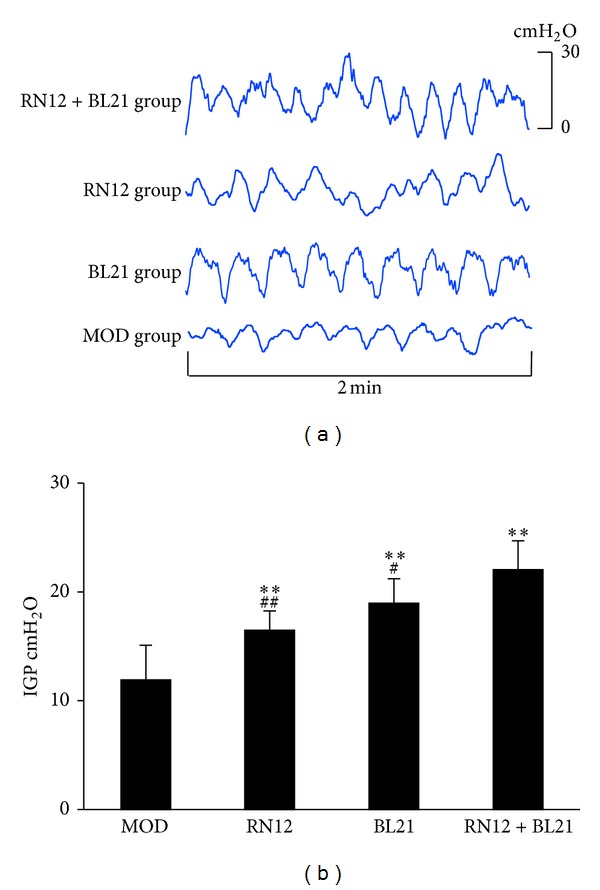
The effects of EA at RN12 and BL21 on IGP. (a) Representative waves of IGP of rats induced by stimulating RN12 and BL21. (b) Summarized data for the effect of stimulation at RN12 and BL21 on intragastric pressure. *n* = 8 for each group, using one-way ANOVA followed by LSD test. **P < 0.01 compared with MOD group; ^##^ *P* < 0.01, ^#^ *P* < 0.05 compared with RN12 + BL21 group.

To determine whether EA at RN12 and BL21 could affect gastric motility in rats, we designed an experiment in which the EA stimulation protocol described above was applied at RN12 and BL21, both alone and in combination. As shown in Figure 1(a), IGP was dramatically increased in the three EA groups, especially in the RN12 + BL21 group (*P* < 0.01, *n* = 8); further, compared with the RN12 and BL21 groups, the increase in IGP was significantly greater in the RN12 + BL21 group (*P* < 0.05, *n* = 8). Thus, these data suggest that EA at RN12 and BL21 can regulate gastric motility, with stimulation of both of these acupoints eliciting a synergistic effect.

### 3.2. The Role of the Vagus in the Regulation of Gastric Motility by EA at RN12 + BL21

**Figure 2:**
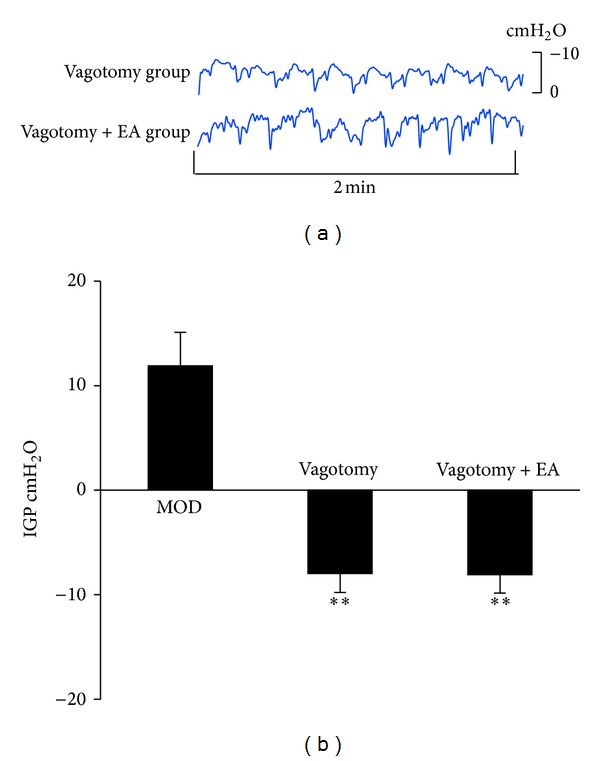
The effects of vagus in regulating IGP by stimulating RN12 + BL21. (a) Representative waves of IGP of rats induced by cutting bilateral subdiaphragmatic vagus with or without stimulating RN12 + BL21. (b) Summarized data for the effect of stimulation at RN12 + BL21 with bilateral subdiaphragmatic vagotomy on intragastric pressure. *n* = 8 for each group, using one-way ANOVA followed by LSD test. **P < 0.01 compared with MOD group.

To determine the role of vagus in mediating the enhancement of gastric motility by EA at RN12 + BL21, we cut the bilateral subdiaphragmatic vagus under an anatomic microscope. We found that the IGP was decreased compared with MOD group (*P* < 0.01, *n* = 8), and the waves were changed. The decreased IGP induced by vagotomy was not restored by EA at RN12 + BL21 (Figures 2(a) and 2(b)). This suggested that the vagus potentially plays a role in mediating the effect of EA at RN12 + BL21 on gastric motility.

### 3.3. The Role of the DVC in the Regulation of Gastric Motility by EA at RN12 + BL21

**Figure 3:**
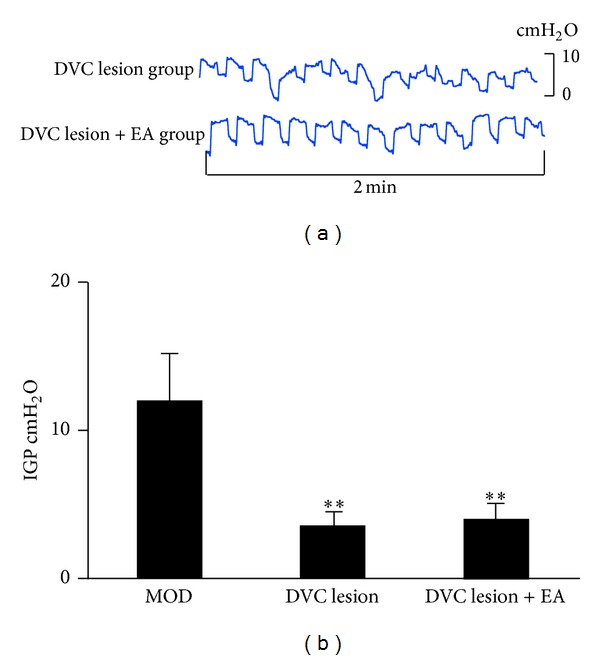
The effects of DVC in regulating IGP by stimulating RN12 + BL21. (a) Representative waves of IGP of rats induced by damaging DVC with or without stimulating RN12 + BL21. (b) Summarized data for the effect of stimulation at RN12 + BL21 with DVC lesion on intragastric pressure. *n* = 8 for each group, using one-way ANOVA followed by LSD test. **P < 0.01 compared with MOD group.

To elucidate whether the DVC was responsible for modulating the effects of EA at RN12 + BL21 on gastric motility, we stereotaxically damaged the DVC with a lesion-making device. We found that this intervention caused IGP to be decreased as compared with the MOD group (*P* < 0.01, *n* = 8), with the waves becoming disordered. The decreased IGP induced by DVC lesion was not restored by EA at RN12 + BL21 (Figures 3(a) and 3(b)). These results suggest that the DVC plays critical roles in regulating gastric motility and that EA at RN12 + BL21 increases gastric motility through the DVC.

### 3.4. EA at RN12 and BL21 Activates DVC Neurons

**Figure 4:**
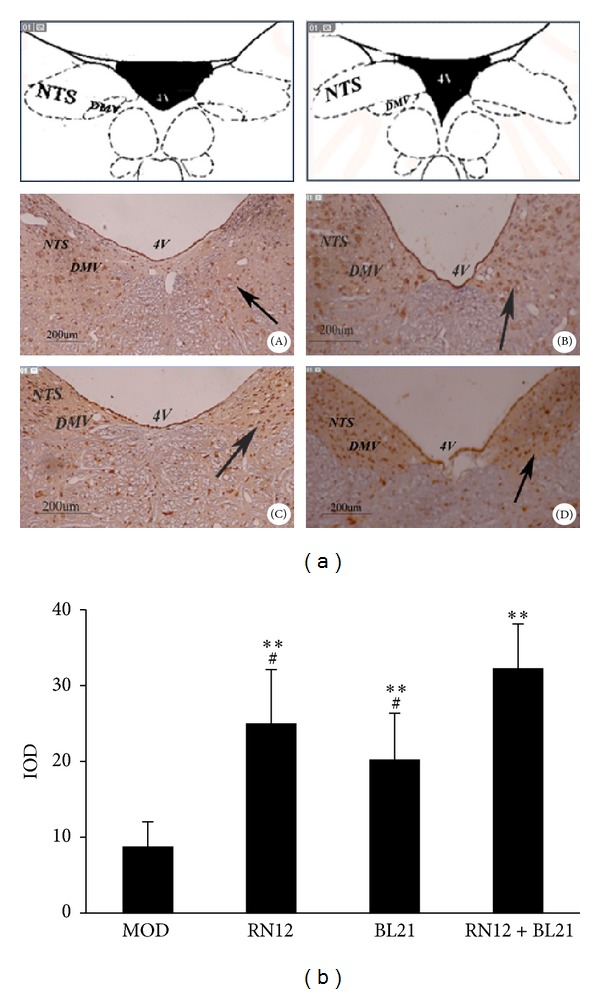
The expression of *c*-*fos* in DVC. (a) Photomicrographs of the medullary sections showing *c*-*fos* immunoreactivity in the DVC (NTS and DMV) in model rats (A) and in rats with EA at RN12 (B), BL21 (C), and RN12 + BL21 (D). The anatomic locations of the photomicrographs are indicated in the top, adapted from the atlas of Paxinos and Watson [30]. *c*-*fos*-positive neurons are presented as dark brown staining in the cell nuclei. Scale bars: 200 *μ*m. 4 V: fourth ventricle. (b) Integral optical density (IOD) of *c*-*fos*-positive neurons in the DVC. Each column represents the mean ± SE of IOD. **P < 0.01 compared with MOD group; ^#^ *P* < 0.05 compared with RN12 + BL21 group.

In the model rats, the number of *c*-*fos*-positive neurons in the DVC was low, while EA markedly induced *c*-*fos* expression in the DVC as compared with the model animals (*P* < 0.01); further, *c*-*fos* expression was statistically increased in the RN12 + BL21 group as compared with the RN12 and BL21 groups (*P* < 0.05) (Figures 4(a) and 4(b)). These findings indicate that the acupuncture signals induced by stimulation of RN12 and BL21 gather in the DVC.

### 3.5. EA at RN12 and BL21 Enhances the Production of Gastrointestinal Hormones in the DVC

Compared with the MOD group, the expression levels of motilin and gastrin in rat DVC were significantly increased in the three EA groups (*P* < 0.05) (Table 1). Our data suggest that gastrointestinal hormones in the DVC participate in the modulation of gastric motility by EA stimulation of RN12 and BL21.

## 4. Discussion

Combined stimulation of the Shu and Mu points is commonly used in clinical practice. The Back-Shu point and Front-Mu point are matched to treat diseases of the viscera and of the bowel. Treatment of hepatopathy requires the selection of BL18 (Shu) with LR14 (Mu), while the treatment of gastropathy necessitates the selection of BL21 (Shu) with RN12 (Mu). While the traditional Chinese medicine literature defines BL21 and RN12 as the points for functional gastrointestinal disorders, insufficient attention has been paid to its effect on these diseases. In this study, we investigated whether EA at RN12 and BL21 could improve gastric motility. We discovered that the combined EA stimulation of gastric Shu and Mu points has synergistic effects. It is worth noting that, although most cases showed an upregulation of gastric motility by EA at BL21 and RN12 [9], in some cases EA at these points failed to accelerate gastric motility, or even inhibited gastric motility, which is similar to previous reports [10].

 With regards to modern anatomy, the nerve segment relationship between internal organs and their Back-Shu and Front-Mu points is very consistent. Modern research shows that there is a specific pathway connecting the stomach with its Back-Shu and Front-Mu points, with visceral and somatic afferent impulses gathering at the spinal cord [11]. A topic of intense investigation is whether or not the convergence is extended to the high centre. On the basis of the acupuncture literature and the modern research of Shu and Mu points combination, the following “targeted convergence” hypothesis has been put forth: the acupuncture signals of Shu and Mu points gather not only in the spinal cord, but also have “targeted convergence” in the brain stem and hypothalamus, achieving integration of high hub through the neural microcircuitry. The essence of Shu and Mu points combination is the convergence of the effector organ and the target. 

 In recent years increasing attention has been paid to studying the interconnections between acupuncture acupoints and brain targets. It has been previously reported that the nervous system, neurotransmitters, and endogenous substances respond to EA [12]. Among these components, the functional activity of the hypothalamus and brainstem is the core foundation of acupuncture-modulated effects [13, 14]. Our finding that EA at RN12 and BL21 could induce the expression of *c*-*fos* in the DVC and that the upregulation in the RN12 + BL21 group was even more marked demonstrates that the acupuncture signals of the gastric Shu and Mu points gather in the medullary DVC. 

 The DVC lies in the dorsomedial part of the medulla oblongata and consists of the NTS and DMV. Sensory inputs from the stomach, both mechanical and chemical, are transmitted mainly to the dorsomedial, medial, and commissural subnucleus of the NTS via the vagus [15]. Both the NTS and DMV play critical roles in regulating gastric activity [16]. The gastric efferent neurons are concentrated in the central portion of the DMV and project to the stomach via gastric branches of the vagus [17]. Laugghton [18] and Semba et al. [19] found that stimulating the DMV and other areas could improve or restrict gastric activity. Some studies showed that the vagal pathway and related DVC potentially play a role in mediating the effect of EA on gastric activities [20–23]. In our study, we found that DVC lesion or bilateral subdiaphragmatic vagotomy caused gastric motility to be decreased, and EA at RN12 + BL21 was not able to restore this decreased gastric motility. It was demonstrated that the DVC was the central target of the gastric Shu and Mu points combination regulating gastric motility, and the effects of the combined stimulation depended upon an intact vagus nerve. 

 Furthermore, a variety of neurotransmitters and neuromodulators (especially neuropeptides) distributed in the central nervous system are involved in regulating gastric function. Gastrointestinal peptides can regulate gastrointestinal motility through the nervous system [24]. In the nervous system, gastrointestinal peptides, such as motilin, can regulate gastrointestinal spontaneous rhythmic contraction [25]. Motilin is a polypeptide composed of 22 amino acids. It has been found in the central and peripheral nervous system of the gastrointestinal wall. The main physiological functions of motilin involve the regulation gastrointestinal motility. We found that EA could promote gastric motility, and this phenomenon might involve the action of motilin and cholecystokinin (CCK) in the periphery [26]. The present study demonstrated that acupuncture could change the level of motilin in bulbus medullae. It has been suggested that acupuncture may regulate level of motilin in bulbus medullae to influence gastric motility [27]. Gastrin is a vital gastrointestinal hormone distributed in the central nervous system and gastrointestinal tract. It acts as a neuromodulator in the central nervous system and as a hormone in gastrointestinal tract, participating in the regulation of gastric motility [28]. It belongs to the family of brain-gut peptides and it promotes gastrointestinal motility. Studies have shown that EA can increase gastrin of the central nervous system and excite the vagal and peptidergic nerves of the peripheral nervous system, with gastrin being one of the important mediators of the effects of acupuncture [29]. Our finding that the expression levels of MTL and GAS in DVC were increased significantly in the three EA groups suggests that gastrointestinal hormones in the DVC participate in EA-regulated gastric motility. 

## 5. Conclusion

Based on the principles of traditional Chinese medicine and with the use of modern technology, this study demonstrated that EA stimulation of the gastric Shu and Mu points can regulate gastric motility, with combined stimulation of these acupoints eliciting a synergistic effect. The effect was closely connected with the DVC, as acupuncture signals generated by EA at gastric Shu and Mu points gather in the DVC, elevating gastrointestinal hormones in the DVC, which in turn play a role in regulating gastric motility through the vagus. Thus, we suggest that the effects of combined EA stimulation of gastric Shu and Mu points may be achieved through the combined efforts of the DVC-vagus nerve-gastric channel.

## Figures and Tables

**Table 1:** Effects of EA on the expression of MTL and GAS in DVC.

Groups	*n*	MTL	GAS
MOD	8	8.20 ± 2.46	13.38 ± 2.87
RN12	8	15.47 ± 3.98*	23.33 ± 4.12*
BL21	8	17.52 ± 3.54*	21.04 ± 3.58*
RN12 + BL21	8	14.68 ± 4.02*	19.92 ± 4.07*

Each value represents the mean ± SE of IOD and the numbers indicated in the table. *P < 0.05 compared with MOD group.
